# Enhanced anti-glioma efficacy of doxorubicin with BRD4 PROTAC degrader using targeted nanoparticles

**DOI:** 10.1016/j.mtbio.2022.100423

**Published:** 2022-09-12

**Authors:** Yihong He, Xin Zan, Junming Miao, Bilan Wang, Yin Wu, Yangmei Shen, Xinchuan Chen, Hongfeng Gou, Songping Zheng, Ning Huang, Yongzhong Cheng, Yan Ju, Xianghui Fu, Zhiyong Qian, Peizhi Zhou, Jiagang Liu, Xiang Gao

**Affiliations:** a Department of Neurosurgery and Institute of Neurosurgery, State Key Laboratory of Biotherapy and Cancer Center, West China Hospital, West China Medical School, Sichuan University and Collaborative Innovation Center for Biotherapy, Chengdu, 610041, China; b Department of Pathophysiology, West China School of Basic Medical Sciences & Forensic Medicine, Sichuan University, 610041, Chengdu, China; c West China Second University Hospital of Sichuan University, Chengdu, 610041, PR China

**Keywords:** Glioma, Resistance, cRGD-PEG-PLA, Doxorubicin, BRD4 PROTAC degrader

## Abstract

Current treatment of glioma is hampered due to the physical blood-brain barrier (BBB) and the resistance to traditional chemotherapeutic agents. Herein, we proposed a combined treatment strategy based on Cyclo (Arg-Gly-Asp-d-Phe-Lys) (cRGDfk) peptides-modified nanoparticle named cRGD-P in a self-assembly method for the co-delivery of doxorubicin (DOX) and BRD4 PROTAC degrader ARV-825 (ARV). Molecular dynamics simulations showed that cRGD-P could change its conformation to provide interaction sites for perfectly co-loading DOX and ARV. The cRGD-P/ARV-DOX exhibited an average size of 39.95 ​nm and a zeta potential of −0.25 ​mV. Increased expression of BRD4 in glioma cells was observed after being stimulated by cRGD-P/DOX, confirming one of the possible mechanisms of DOX resistance and the synergistic tumor inhibition effect of BRD4 degrading ARV combined with DOX. In the study, the combination of DOX and ARV in the cRGD-P nanoparticle system exhibited synergistic suppression of tumor growth in glioma cells on account of cell cycle arrest in the G2/M phase and the activation of tumor cells apoptosis-related pathways including triggering caspase cascade and downregulating Bcl-2 as well as upregulating Bax. The cRGD-P/ARV-DOX system could effectively suppress the heterotopic and orthotopic growth of glioma by increasing tumor apoptosis, inhibiting tumor proliferation, and decreasing tumor angiogenesis in vivo. Therefore, the cRGD-modified nanoparticle to co-deliver DOX and ARV provides a potential platform for exploiting a more effective and safer combination therapy for glioma.

## Graphical abstract

Schematic representation of the structure model about co-loading DOX and ARV-825 by cRGD-decorated nanoparticles (cRGD-PEG-PLA), and the processes of drug delivery in orthotopic glioma model.

**Figure:**
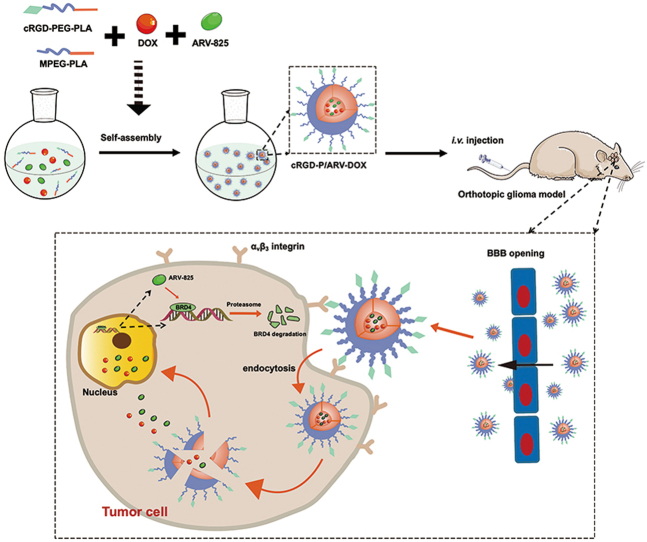

## 1 Introduction

Glioma is the most frequent malignant brain tumor, accounting for 75% of cases in grow-ups, with an immensely high recurrence rate and mortality [1]. To date, the 5-year relative survival of glioma patients was less than 5% [2]. At present, surgery, chemotherapy and radiotherapy are the predominant treatment strategies [3]. But infiltration features of glioma and the existence of blood-brain barrier (BBB) lead to incomplete tumor resection and difficulty for drugs to reach the tumor site [4,5]. Chemotherapy drugs, such as doxorubicin (DOX), have the superior activity to kill cancer cells, but glioma cells still develop some epigenetic changes to survive through drug resistance [6,7]. Drug resistance induced by the epigenetic changes on tumor cells can hardly be surmounted [8]. Moreover, the increased dose of chemotherapy drug for achieving sufficient anti-tumor efficacy could lead to some severe side effects [8]. Therefore, the combination of DOX and some drug resistance-related inhibitors may have potentially synergistic effects for the treatment of glioma.

A promise has been focused on the modulation of epigenetic [9]. One classical example is the targeted suppression of the bromodomain and extra-terminal domain (BET) including bromodomain-containing protein 4 (BRD4), which displays superiority of inhibiting tumor growth. BRD4 is an important epigenomic reader of gene transcriptional regulation [10,11]. The overexpression of BRD4 in cancer cells can induce abnormal expression of its downstream genes involving some pivotal oncogenes such as c-Myc and Bcl-2, which contributes to the occurrence and development of glioma, acute myeloid lymphoma (AML), breast cancer, and other tumors [[12], [13], [14], [15]]. Therefore, BRD4 may work as a potential therapeutic target for drug-resistant cancers. A molecule here that has attracted our attention is ARV-825 (ARV) that used the PROTACs (proteolysis targeting chimeras) technology to induce BRD4 ubiquitylation by the E3 ligases, resulting a sustained and profound depletion of BRD4 proteins by proteasome [[16], [17], [18]]. Although small-molecule BRD4 inhibitors such as iBET, JQ1 and OTX015 have shown their preclinical anti-cancer potential by preventing the binding of BRD4 with acetylated chromatin, their lack of sustained inhibition leads to re-accumulation of BRD4 protein and limited suppression of key oncogenes and potentially inducing resistance [[19], [20], [21]]. Therefore, ARV has been considered a charming agent to overcome drug resistance.

DOX has an obvious adverse effect to cause injury to normal cells such as the mitochondria-rich cardiomyocyte. As the cytotoxic effect is not cancer cells-selective, targeted delivery is adopted to decrease systemic toxicity. Moreover, the limited effects of DOX lie in the poor bioavailability, short half-life as well as failure to cross the BBB [22]. Meanwhile, PROTACs have aqueous solubility and permeability problems [23]. In addition, Rathod et al. showed that ARV-825 is quickly metabolized by the drug-metabolizing enzyme CYP34A [24]. To address this issue, nanotechnology has been an attractive strategy. Multifarious nanoparticles have been generally applied as drug delivery vector to target the tumor with low systemic toxicity for brain tumors therapy [25,26]. Especially, some nanoparticles attempted to increase water solubility, and enhanced the bypass of BBB for the deep penetration of chemotherapy drug to glioma locus [[27], [28], [29], [30]]. Integrins are used as natural targets of receptor-mediated endocytosis [31]. α_v_β_3_ integrin is prominently expressed in tumor tissues, while limited or even absent in normal tissues, becoming a potential target for drug delivery [32,33]. It is reported that the binding ability of Cyclo (Arg-Gly-Asp-d-Phe-Lys) (cRGDfk) and α_v_β_3_ integrin is highly specific and stable and cRGD-modified nanoparticles showed priority to increase cellular uptake and targeted property [34,35]. Therefore, α_v_β_3_ integrin-targeted therapy is a potential method to improve the treatment of glioma.

**Scheme 1:**
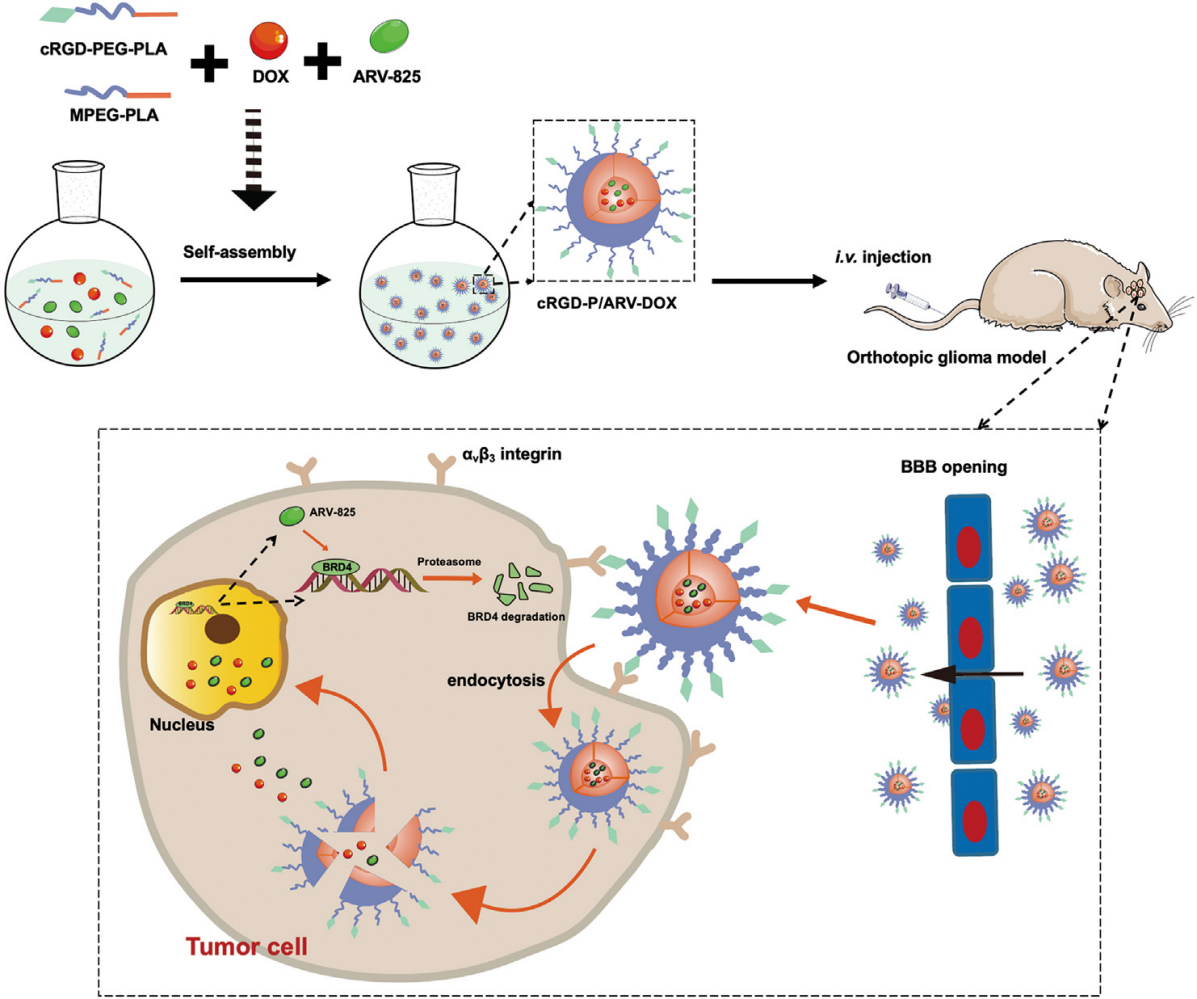
Schematic illustration of the system co-loading DOX and ARV-825 by cRGD-decorated nanoparticles, and the processes of drug delivery in orthotopic glioma model.

Herein, we devised a novel targeted drug delivery system with cRGD-modified poly (ethylene glycol)- poly(lactide) (cRGD-PEG-PLA) by a self-assembly procedure to co-deliver DOX and ARV (cRGD-P/ARV-DOX). Scheme 1 showed our design of tumor-targeting nanoparticles loading dual drugs to cross the BBB and reach tumor sites for inducing a synergetic effect against tumors. In the study, we examined the therapeutic efficacy against glioma both in vitro and in vivo. Moreover, the underlying anti-tumor mechanisms and safety of this nanodrug were investigated. Our findings indicated that DOX and ARV delivered concurrently by cRGD-P could improve anti-cancer effects through overcoming acquired drug-resistance, suggesting promising application in glioma therapy as well as other cancer therapies.

## 2 Materials and methods

### *2.1* Synthesis of nanocomposite

The MPEG_2000_-PLA_2000_ polymer was prepared by ring opening of DL-Lactide that was activated by MPEG_2000_. Shortly, MPEG_2000_ (5.0 ​g) and DL-lactide (5.0 ​g) were together added into a dry round-bottomed flask under nitrogen in mild agitation. Then 0.05 ​g catalyzer Sn (Oct)_2_ was dropped into the reaction system when the temperature was up to 140 ​°C for 8 ​h. Next, the obtained product was dissolved in ddH_2_O and transferred into a dialysis bag (MWCO ​= ​3500 ​Da) for further purification. Finally, they were freeze-dried for 5 days to gain powdery MPEG_2000_-PLA_2000_.

The cRGD_fk_-PEG_2000_-PLA_2000_ polymer was successfully synthesized in our study. Firstly, PLA_2000_-COOH (500 ​mg) was melted in trichloromethane (10 ​mL). Then the solution was added DIC (2.0 eq) and triethylamine (2.0 eq), followed by the addition of HOOC-PEG_2000_-NH_2_ (2.0 eq) in chloroform, by continuous stirring for 6 ​h at room temperature. After being processed by rotary evaporation, the acquired solution was dialyzed (MWCO ​= ​3500 ​Da) and lyophilized to obtain HOOC-PEG_2000_-PLA_2000_. Subsequently, they were melted in NMP and added EDC (2.0 eq) and NHS (2.0 eq). After reacting 2 ​h at room temperature, the cRGD_fk_ (1.2 eq) and triethylamine (3.0 eq) were poured in the above compound and the reaction was stirred at room temperature overnight. The obtained cRGD-PEG-PLA copolymer was further dialyzed (MWCO ​= ​3500 ​Da). Finally, the ^1^H NMR was applied to confirm the structure of the synthetic cRGD-PEG-PLA.

MPEG-PLA loading single ARV (M/ARV), MPEG-PLA loading single DOX (M/DOX), MPEG-PLA loading both ARV and DOX (M/ARV-DOX) were constructed by a self-assembly method. Firstly, 90 ​mg of MPEG_2000_-PLA_2000_ and 5 ​mg of ARV were respectively introduced into 2 ​mL acetone, then the solvent was dried to form a film through rotary evaporation at 55 ​°C. Then the dispersed film was dissolved in 2 ​ml water for hydration, forming a core-shell-structured M/ARV. Next, 7 ​ml of M/ARV micelles and 1 ​ml of phosphate-buffered solution (10 ​× ​, pH 7.4) were mixed with stirring. Then 2 ​ml of DOX (2.5 ​mg/ml) was introduced into them for 20 ​min incubation to form the M/ARV-DOX. M/DOX was synthesis by self-assembly method, on account of low solubility of DOX in phosphate-buffered solution at pH 7.4, being packaged by the hydrophobic core of the MPEG-PLA micelle. The complex cRGD-PEG_2000_-PLA_2000_/ARV-DOX (cRGD-P/ARV-DOX) was prepared using same method by adding ARV (5 mg), DOX (5 mg), MPEG_2000_-PLA_2000_ (67.5 mg) and cRGD-PEG_2000_-PLA_2000_ (22.5 mg). The morphology of cRGD-P/ARV-DOX was observed by transmission electron microscopy (TEM, Hitachi H600, Japan). The dynamic light scattering (DLS) method was used to learn the average size and zeta potential of the complex by a Zetasizer NanoZS (Malvern Instruments, Ltd., Worcestershire, UK).

### *2.2* Computational simulation between cRGD-PEG-PLA, DOX and ARV

The two-dimensional structures of cRGD-PEG-PLA, DOX and ARV were firstly devised using ChemDraw and then transformed into three-dimensional structures by Chem3D. Next, ARV and DOX were positioned around the cRGD-PEG-PLA polymer. LAMMPS software was used to simulate the molecular dynamics of the cRGD-PEG-PLA/ARV-DOX complex in aqueous circumstances (pH ​= ​7.0) for a total of 10 ns. The snapshot of conformation about the complex was recorded every 2 ns to analyze the interaction method.

### *2.3* Cell viability assay

Cytotoxicity of cRGD-P/ARV, cRGD-P/DOX and cRGD-P/ARV-DOX was measured by MTT assay. The prepared drug micelles were diluted to different concentrations at 0, 0.0078, 0.0156, 0.0312, 0.0625 ​μg/ml by culture medium. GL261 cells were seeded at a density of 3 ​× ​10^3^ and 1.5 ​× ​10^3^ ​cells per well of 96-well plates overnight. Then the supernatant was displaced with the prepared samples for 24 or 48 ​h incubation. U251 cells were treated with relative high concentrations (0.0625, 0.125, 0.25 ​μg/ml). At last, MTT (5 ​mg/ml) was added for another 2 ​h incubation with cells. After discarding the supernatant, each well was added into 150 ​μl of DMSO. At last, the cell viability was determined with a microplate reader (OPTImax, Molecular Dynamics).

### *2.4* Cell cycle analysis

Cells were firstly plated in culture dishes overnight. After that the supernatant was removed and then replaced by serum-free DMEM medium for 12 ​h. GL261 cells were incubated with cRGD-P/ARV, cRGD-P/DOX and cRGD-P/ARV-DOX at 0.0625 ​μg/ml for 24 ​h (U251 at 0.125 ​μg/ml for 48 ​h). The collected single-cell suspension was washed with pre-cooled PBS and fixed in 70% ethanol at 4 °C for 24 ​h. Afterwards, the cells were stained by PI containing RNase and analyzed by an ACEA NovoCyteTM (Novo Express International, Inc, USA) ﬂow cytometer at a minimum speed.

### *2.5* Cell apoptosis assay

GL261 ​cells were treated with cRGD-P/ARV, cRGD-P/DOX or cRGD-P/ARV-DOX at the concentrations of 0, 0.0625, 0.125, 0.25, 0.5 ​μg/ml for 24 ​h. U251 cells were treated with cRGD-P/ARV, cRGD-P/DOX or cRGD-P/ARV-DOX at 0, 0.0625, 0.125, 0.25 ​μg/ml for 48 ​h. Subsequently, the obtained single-cell suspension was stained by an Annexin V-FITC/PI apoptosis detection kit (BD Pharmingen). Flow cytometric analysis was employed to determine the proportions of necrotic and apoptotic cells.

### *2.6* Anti-tumor effects in subcutaneous glioma models

Harvested GL261 ​cells were washed and then resuspended with pro-cooled PBS to obtain the concentration of 5 ​× ​10^6^ ​cells/ml. Mice were subcutaneously inoculated with 200 ​μl of the cell suspension. After 7 days, these mice were randomly allocated into six groups and intravenously injected with the following therapeutic schemes: (A) GS: 5% glucose solution, (B) Vehicle: cRGD-P, (C) M/ARV, (D) M/DOX, (E) M/ARV-DOX and (F) cRGD-P/ARV-DOX. The injected solution per mice was 200 ​μl volume and the dosages of M/ARV and M/DOX both are 2.5 ​mg/kg body weight of mice. Moreover, mice were treated every three days for consecutive 3 times. During the treatment, the xenograft volume and mice weight were recorded every other day. Tumor volume was calculated based on the following formula:

Tumor volume ​= ​0.52 ​× ​Length ​× ​Width^2^

On the 16th day after inoculation, blood sample was obtained through eyeball extraction. Then mice were euthanized, and the tumor tissues of each mice were harvested and photographed, as well as the tumor weights were recorded. Vital organs were carefully isolated for further study.

### *2.7* Anti-tumor effects in an orthotopic implantation glioma model

To establish orthotopic implantation glioma model, 1×10^5^ GL261-Luc cells expressing the transfected luciferase gene were suspended in 4 ​μl serum-free DMEM and kept on ice until orthotopic implantation. After being anesthetized by isoflurane using a rodent anesthesia system (RWD, USA), the mice were fixed with digital stereotactic apparatus (RWD, USA) and cut the skin along the midline of the head with a surgical blade to expose the cranium. The entry point was the former fontanelle as the origin, 2.5 ​mm to the right, and 0.5 ​mm backward. Then the needle was inserted into 4.5 ​mm vertically and retreated another 1 ​mm to leave space for intake of tumor suspension. Then the GL261-Luc cells were injected into the intracranial cavity by a 10 ​μl Hamilton micro-syringe (Hamilton, Darmstadt, Germany). After injection at a certain speed, the needle stayed for 5 ​min before pulling out the micro-syringe. Then the injection site was blocked by bone wax and the skin was sutured with absorbable stitches.

Three days post implantation, mice were treated with different drugs through intravenous injection every three days for 3 times. The mice body weight was monitored during the treatments. At last, mice were anesthetized by isoflurane and then intraperitoneally injected with luciferase substrates to obtain the bioluminescence imaging pictures by the IVIS Lumina Imaging System (Caliper, USA). Then, all animals were euthanized, and brain tissues of different groups were fixed in paraformaldehyde for H&E staining to assess the effect of drugs in vivo ulteriorly.

### *2.8* TUNEL assay

TUNEL assay labelling apoptotic cells was applied to measure the pro-apoptotic effect of drug. Tumor tissues from the subcutaneous model of glioma were soaked into formalin and embedded in paraffin, then sliced up for TUNEL staining, which was conducted according to the TUNEL staining kit (Promega, USA). Stained sections were observed by the fluorescence microscope (Leica, Germany). The TUNEL staining positive cells were considered as apoptotic cells.

### *2.9* Immunohistochemical analysis

Subcutaneous tumors sections of each group were prepared as above. For evaluating the anti-angiogenesis and anti-proliferation effects of cRGD-P/ARV-DOX in vivo, these sections from each group were stained with CD31 or Ki67 antibodies and photographed by the optical microscope (Leica, Germany). Five regions were counted to calculate CD31 positive vessels and Ki67 positive cells.

### *2.10* Safety assessment

After the last treatment, blood of mice in the subcutaneous GL261 tumor model was harvested and placed in a 4 ​°C refrigerator overnight, then centrifugated at 4 ​°C for 15 ​min to obtain serum. The serum was further used for serological biochemistry analysis by an automatic analyzer Cobas C311 (Roche, Switzerland). Vital organs (heart, liver, spleen, lung and kidney) of mice receiving different treatments were sliced for H&E staining.

### *2.11* Statistical analysis

The GraphPad Prism software was exploited for statistical analysis. As for two groups comparison, the Student's *t*-test was used. As for multiple groups comparisons, the ordinary one-way analysis of variance (one-way ANOVA) was used. All statistical values were tested at least three independent experiments and presented as mean ​± ​standard error of measurement (SEM). All comparisons with P ​< ​0.05 were considered statistically significant.

## 3 Results

### *3.1* Preparation and characteristics of cRGD-P/ARV-DOX

The preparation of MPEG-PLA was introduced in Fig. S1. The procedure of synthesis of cRGD-PEG-PLA were listed in Fig. S2A, and chemical structure was further verified by ^1^H NMR (Fig. S2B). The peaks at 3.50 ​ppm (a) belonging to the PEG segment, and the peaks at 5.18 (b), 1.46 (c) ppm connected with PLA block, as well as the peaks of cRGD at 7.23 and 7.17 ​ppm (d), 7.64–8.40 ​ppm (e ​+ ​f) were all detected in the ^1^H NMR of cRGD-PEG-PLA.

**Fig. 1:**
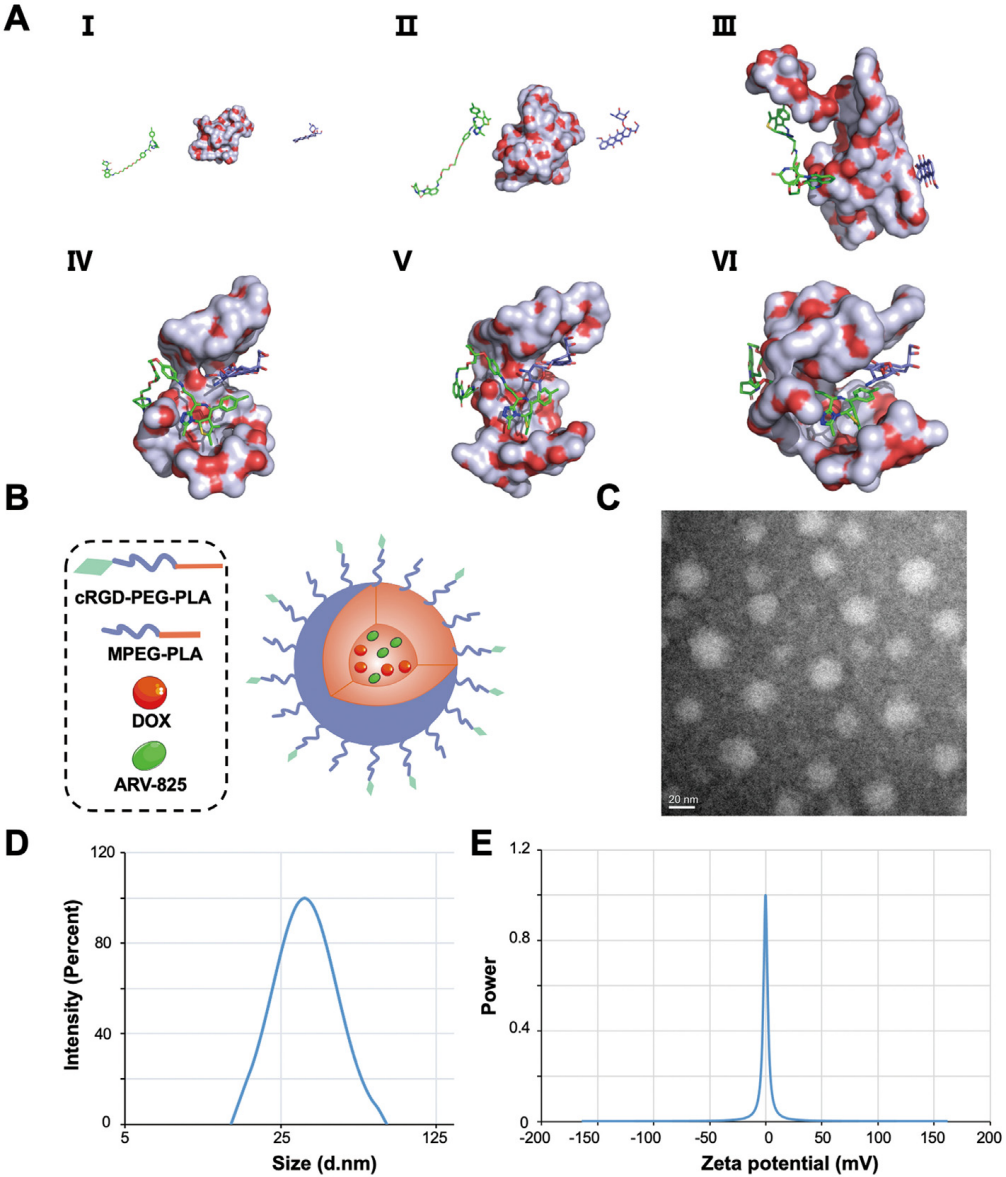
Characteristics of cRGD-P/ARV-DOX micelles. (A) The molecular dynamics simulation of the interactions among cRGD-PEG-PLA, DOX and ARV in the water environment (pH ​= ​7). (Ⅰ) The initial conformation of cRGD-PEG-PLA, DOX and ARV. The left, the middle and the right graphics represent ARV, cRGD-PEG-PLA and DOX, respectively. The conformations Ⅱ), Ⅲ), Ⅳ), Ⅴ) and Ⅵ) corresponded to the snapshots of this complex at 2, 4, 6, 8, and 10 ns, respectively. (B) Structural diagram of cRGD-P/ARV-DOX. (C) TEM image of cRGD-P/ARV-DOX. (D) Particle size of cRGD-P/ARV-DOX. (E) Zeta potential of cRGD-P/ARV-DOX.

cRGD-P/ARV-DOX complex was synthesized by a self-assembly way for co-encapsulation of DOX and ARV. The interplays among cRGD-PEG-PLA, DOX and ARV in the water (pH ​= ​7) were studied by molecular dynamics simulation. They constantly changed their position and conformation to reach a stable state, by which DOX and ARV found an interaction site to link with cRGD-PEG-PLA and were well encapsulated by cRGD-PEG-PLA (Fig. 1A and Fig. S3). The core-shell structure of cRGD-P/ARV-DOX was introduced in Fig. 1B. Meanwhile, the micelles exhibited a spherical structure and was no significant difference in its morphology by TEM (Fig. 1C). The average diameter of cRGD-P/ARV-DOX was 39.95 ​nm, with a near electroneutral zeta potential of −0.25 mv (Fig. 1D and E). The result showed that the small average diameter of cRGD-P/ARV-DOX could be an ideal drug delivery system for glioma therapy.

### *3.2* Increased cytotoxicity and cell cycle arrest in vitro

**Fig. 2:**
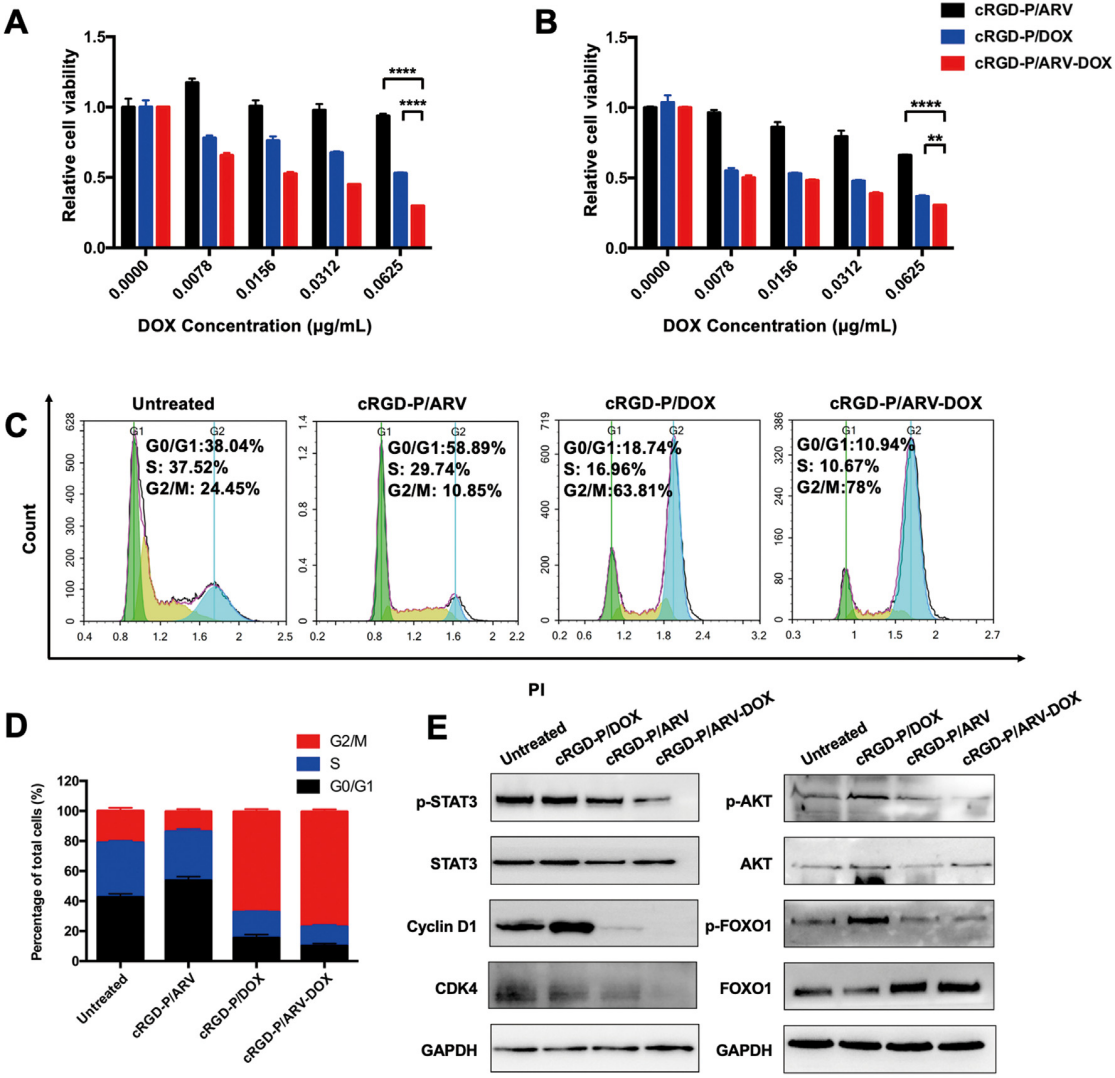
Synergistic effects of DOX and ARV in the cRGD-P carrier system on cytotoxicity and cell cycle arrest in GL261 ​cells. MTT assay was used to assess cytotoxicity of cRGD-P/ARV, cRGD-P/DOX and cRGD-P/ARV-DOX from 0 ​μg/ml to 0.0625 ​μg/ml at 24 ​h ​(A) and 48 ​h ​(B). n ​= ​3. (C) Flow cytometric analysis. GL261 cells were treated by cRGD-P/ARV, cRGD-P/DOX and cRGD-P/ARV-DOX at 0.0625 ​μg/ml for 24 ​h. (D) Populations of cells at G0/G1, S and G2/M phases were displayed as percentages of the whole cell population. (E) Western blotting was performed to detect the cell proliferation and cycle-related proteins. All gene expressions were normalized to GAPDH (reference gene).

We first investigated the expression level of BRD4 after being stimulated by cRGD-P/DOX in GL261 ​cells and found that the expression of BRD4 was rapidly elevated within 12 ​h that was confirmed by protein analysis (Fig. S4). The MTT assay showed that the use of DOX obviously impaired the growth of GL261 ​cells, while cRGD-P/ARV showed a relatively slight inhibitory effect to GL261 ​cells. Moreover, compared with cRGD-P/DOX, cRGD-P/ARV-DOX has greater cytotoxicity to GL261 ​cells at 24 ​h and 48 ​h (Fig. 2A and B). When the concentration of them was both 0.0625 ​μg/ml, the cytotoxic effect for GL261 ​cells is even pronounced (cRGD-P/ARV-DOX vs cRGD-P/DOX, P ​< ​0.01). Analogously, these results were discovered in U251 ​cells and the concentration of 0.1250 ​μg/ml in cRGD-P/ARV-DOX group showed better synergetic anti-cancer effects at 48 ​h (Fig. S5). These results displayed the sensitizing effect of ARV-825 to DOX. In line with our speculation, cRGD-P/DOX-induced BRD4 expression is responsible for DOX resistance, and the BRD4 degrader ARV-825 could relieve drug resistance to improve the cytotoxic effect of DOX in the treatment of cancer.

For exploring the possible reasons for inhibition of glioma proliferation by the combination therapy, the cell cycle and associated proteins were investigated. When GL261 ​cells were treated with cRGD-P/ARV, the cell cycle analysis result displayed that the percentage of cells in the G0/G1 phase had an increase while that in the S phase had a slight decrease (Fig. 2C and D). cRGD-P/DOX greatly elevated the percentage of cells in the G2/M phase while reduced cells in the S phase by nearly 20%, which reflected an effect on the transformation from the S to G2/M phase. As cells were treated with cRGD-P/ARV-DOX, the percentage of cells in the S phase reduced to the lowest and most were arrested in the G2/M phase. Similarly, these results were also revealed in U251 ​cells (Figs. S6A and B). Treatment with cRGD-P/ARV-DOX in GL261 ​cells and U251 ​cells suppressed the activation of STAT3 and AKT and led to the downregulation of p-FOXO1/FOXO1, cyclin D1 and its partner kinase CDK4 (Fig. 2E and Fig. S8A). Thus, these results suggested that G2/M phase was blocked by cRGD-P/ARV-DOX, resulting the inhibition of cell proliferation.

### *3.3* Promoted cell apoptosis in vitro

**Fig. 3:**
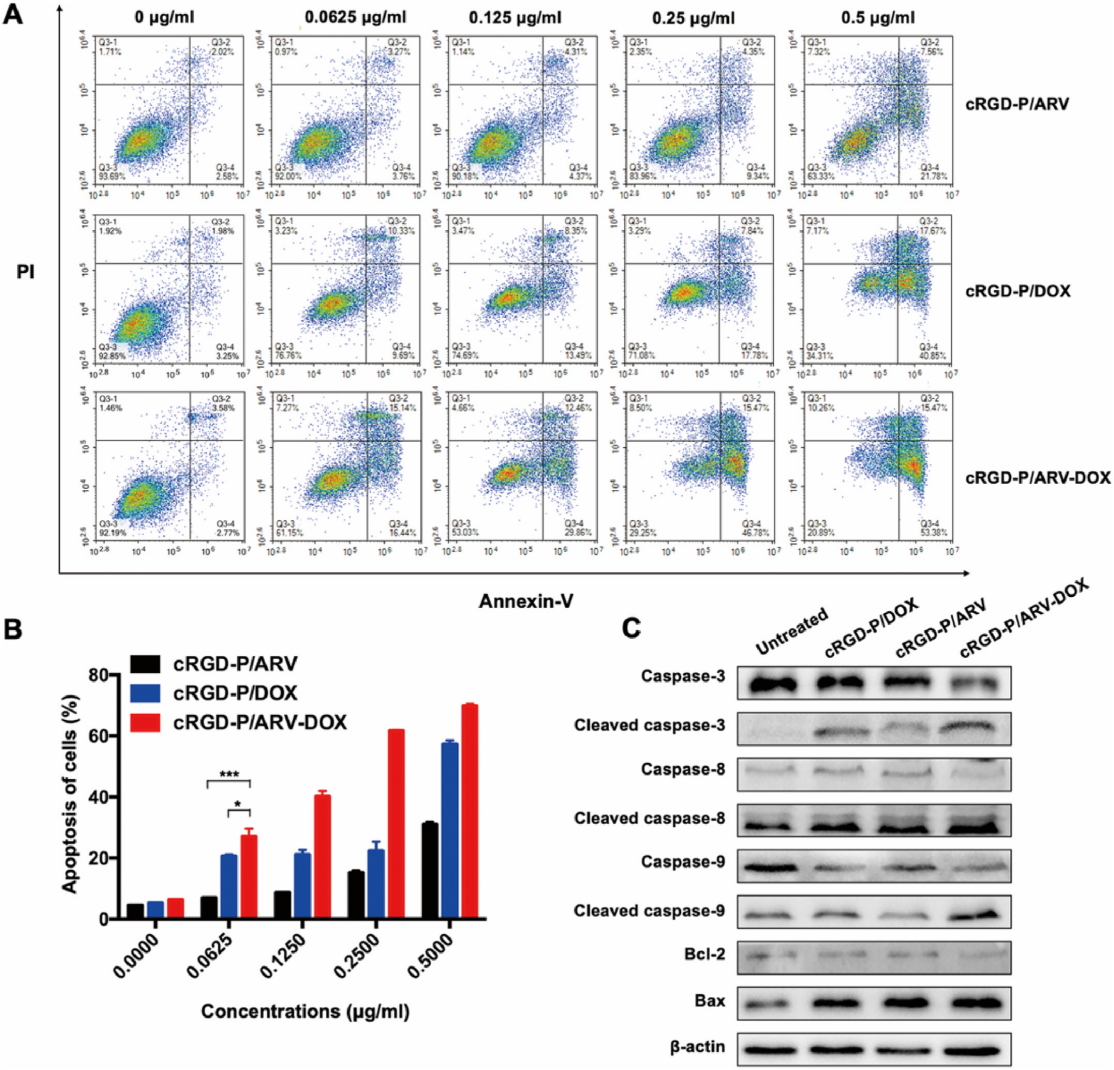
Synergistic effects of DOX and ARV in the cRGD-P carrier system on tumor cell apoptosis. GL261 cells were treated with cRGD-P/ARV, cRGD-P/DOX and cRGD-P/ARV-DOX at concentrations of 0 ​μg/ml, 0.0625 ​μg/ml, 0.125 ​μg/ml, 0.25 ​μg/ml and 0.5 ​μg/ml for 24 ​h. (A)(B) Cells were collected and stained with Annexin-V and PI for flow cytometric analysis. (C) Western blot of apoptosis-related proteins. GL261 cells were exposed to cRGD-P/ARV, cRGD-P/DOX and cRGD-P/ARV-DOX at 0.0625 ​μg/ml for 24 ​h. All gene expressions were normalized to β-actin (reference gene).

The effect of cRGD-P/ARV-DOX on apoptosis were examined by Annexin-V/PI flow cytometry. Results showed that cRGD-P/ARV induced inconspicuous effects at the concentrations from 0 ​μg/ml to 0.25 ​μg/ml, suggesting that single therapy with cRGD-P/ARV at low concentration after 24 ​h exposure has little effect on GL261 ​cells apoptosis. The percentage of apoptotic cells in the cRGD-P/ARV-DOX group was prominently more than those in the cRGD-P/ARV group and cRGD-P/DOX group (Fig. 3A and B). Analogously, the synergistic pro-apoptosis effect was observed in U251 ​cells (Figs. S7A and B).

For further studying the possible mechanism, apoptosis-related proteins were detected. Compared with the cRGD-P/ARV or cRGD-P/DOX group, caspase-3 has shown a more pronounced activation in the cRGD-P/ARV-DOX group. our research also suggested that cRGD-P/ARV-DOX induced the activation of caspase-8, -9, which contributed to the regulation of external and internal apoptosis pathways. Moreover, results in our study showed obvious downregulation of Bcl-2 and upregulation of Bax in GL261 and U251 ​cells after being treated with cRGD-P/ARV-DOX (Fig. 3C and Fig. S8B). These protein expressions were normalized to β-actin or GAPDH (reference gene). Therefore, the cRGD-P/ARV-DOX micelles promoted apoptosis of glioma cells by activating the caspase cascade, down-regulating Bcl-2 expression and up-regulating BAX expression.

### *3.4* Anti-tumor efficacy in subcutaneous xenograft model

**Fig. 4:**
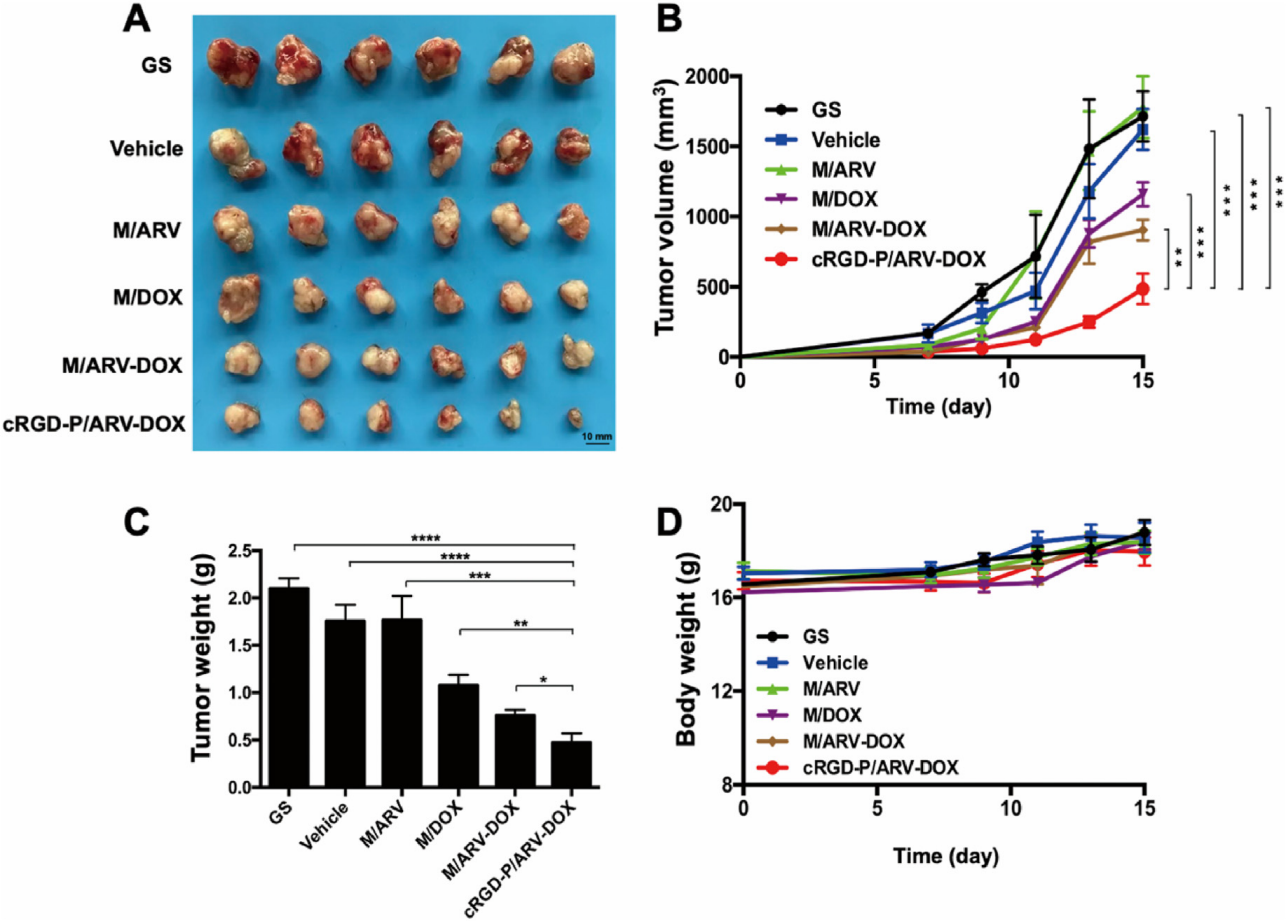
Combination therapy with DOX and ARV by cRGD-modified nanoparticle delivery system improved the anti-tumor activity in subcutaneous GL261 model. C57BL/6 mice were subcutaneously inoculated with 1 ​× ​10^6^ GL261 ​cells and received with following treatments by intravenous injection: GS; Vehicle; M/ARV; M/DOX; M/ARV-DOX or cRGD-P/ARV-DOX. (A) Photos of the tumors from different groups. (B) Tumor growth curves. (C) Tumor weights. (D) Mice body weights. (n ​= ​6; ∗*p* ​< ​0.05, ∗∗*p* ​< ​0.01, ∗∗∗*p* ​< ​0.001, ∗∗∗∗*p* ​< ​0.001).

We further assessed the anti-cancer activity of DOX and ARV co-delivered by cRGD-modified nanoparticle in a GL261 subcutaneous xenograft model. According to the tumor growth curves, average tumor size and tumor weight at the end of treatment, there was a superior retardation effect of tumor growth in the cRGD-P/ARV-DOX group compared with other groups (Fig. 4A–C). Especially, when compared with the non-targeted combination therapy, the tumor-targeting delivery by cRGD-P further enhanced the therapeutic effects against GL261 subcutaneous tumor. No obvious change in body weight was found among all the groups (Fig. 4D). Taken together, the co-delivery of DOX and ARV by cRGD-modified nanoparticles could induce an apparent effect in suppressing glioma growth.

### *3.5* Anti-tumor efficacy in an orthotopic glioma model

**Fig. 5:**
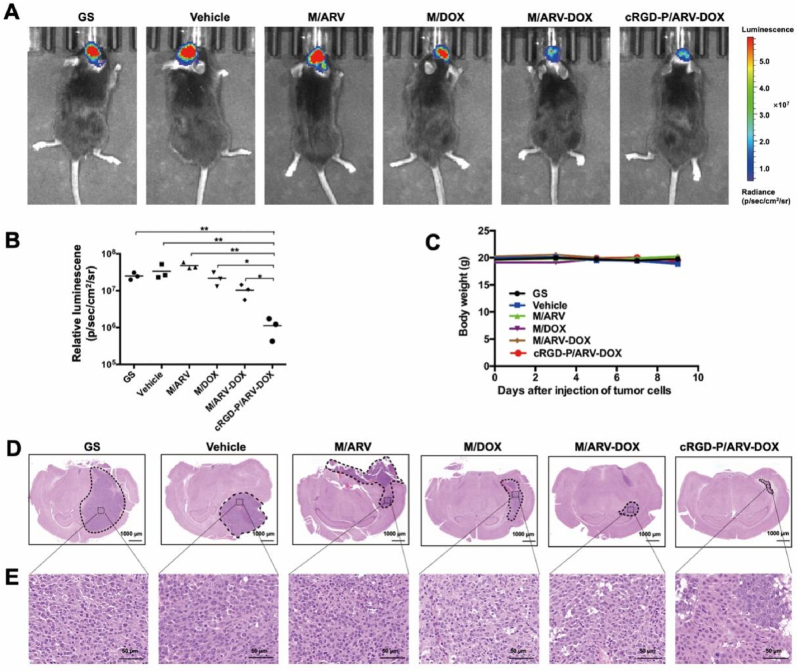
Combination therapy with DOX and ARV by cRGD-modified nanoparticle delivery improved anti-tumor activity in an orthotopic implantation glioma model. C57BL/6 mice were intracranially inoculated with 1 ​× ​10^5^ GL261-Luc cells and received with following treatments by vein injection: GS; Vehicle; M/ARV; M/DOX; M/ARV-DOX or cRGD-P/ARV-DOX. (A) Optical in vivo imaging. (B) Quantification of the bioluminescent signal intensity. (C) Body weights (n ​= ​5). (D) H&E staining of the brain. (E) Enlargement of the corresponding position of the tumor.

The synergistic anti-cancer effect was ulteriorly evaluated in an orthotopic glioma model in mice. In this study, the luciferase-transfected GL261 ​cells were used. In Fig. 5A and B, the cRGD-P/ARV-DOX group showed the weakest bioluminescent signal intensity in the tumor site. Bodyweight curves showed no obvious change in the different groups (Fig. 5C). It was clearly seen that the minimum tumor volume in brain tissues from cRGD-P/ARV-DOX group by the eosin and hematoxylin (H&E) staining (Fig. 5D and E). Specially, the cRGD-modified nanoparticles were able to enhance the anti-tumor efficacy compared to non-modified ones. This strategy achieved better curative effects. Therefore, co-administration of DOX and ARV by cRGD-modified nanoparticle delivery had obvious therapeutic effects against the tumor progression and growth in vivo.

### *3.6* Promotion of cell apoptosis in vivo

**Fig. 6:**
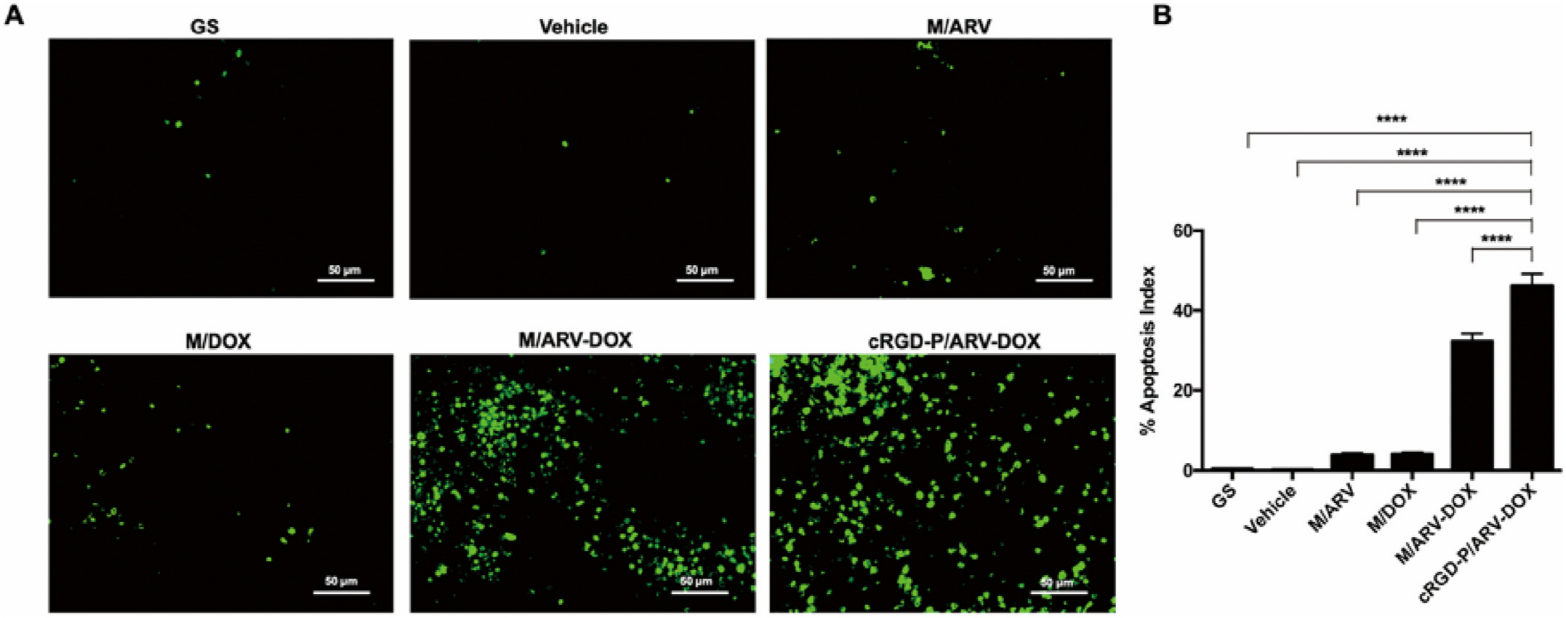
Combination therapy with DOX and ARV by cRGD-modified nanoparticle delivery promoted tumor cell apoptosis in the subcutaneous GL261 glioma model. (A) TUNEL assay was applied to detect cell apoptosis in tumor sections. The photos presented randomly selected field in tumor sections from different groups. (B) The average percentage of TUNEL-positive cells in each group was counted and analyzed. (Mean ​± ​SEM, n ​= ​5, ∗∗∗∗*p* ​< ​0.001).

Next, we estimated the intratumoral apoptosis in the GL261 subcutaneous xenograft model. After three times treatments, tumor sections were stained by TUNEL staining to study the pro-apoptotic effect of cRGD-P/ARV-DOX. The average apoptotic index was 0.41% in the GS group, 0.22% in the vehicle group, 3.94% in the M/ARV group, 4.13% in the M/DOX group, 32.33% in the M/ARV-DOX group and 46.16% in the cRGD-P/ARV-DOX group (Fig. 6A and B). When compared with cRGD-P/ARV, cRGD-P/DOX or non-targeted combination therapy, the cRGD-P/ARV-DOX group showed more fluorescence signals (green color), which marked the apoptosis cells. The group treated with cRGD-P/ARV-DOX micelles displayed the most apoptotic cells, demonstrating that the combination therapy by cRGD-modified nanoparticles significantly improved anti-tumor activity by inducing cells apoptosis.

### *3.7* Inhibition of cell proliferation and angiogenesis in vivo

**Fig. 7:**
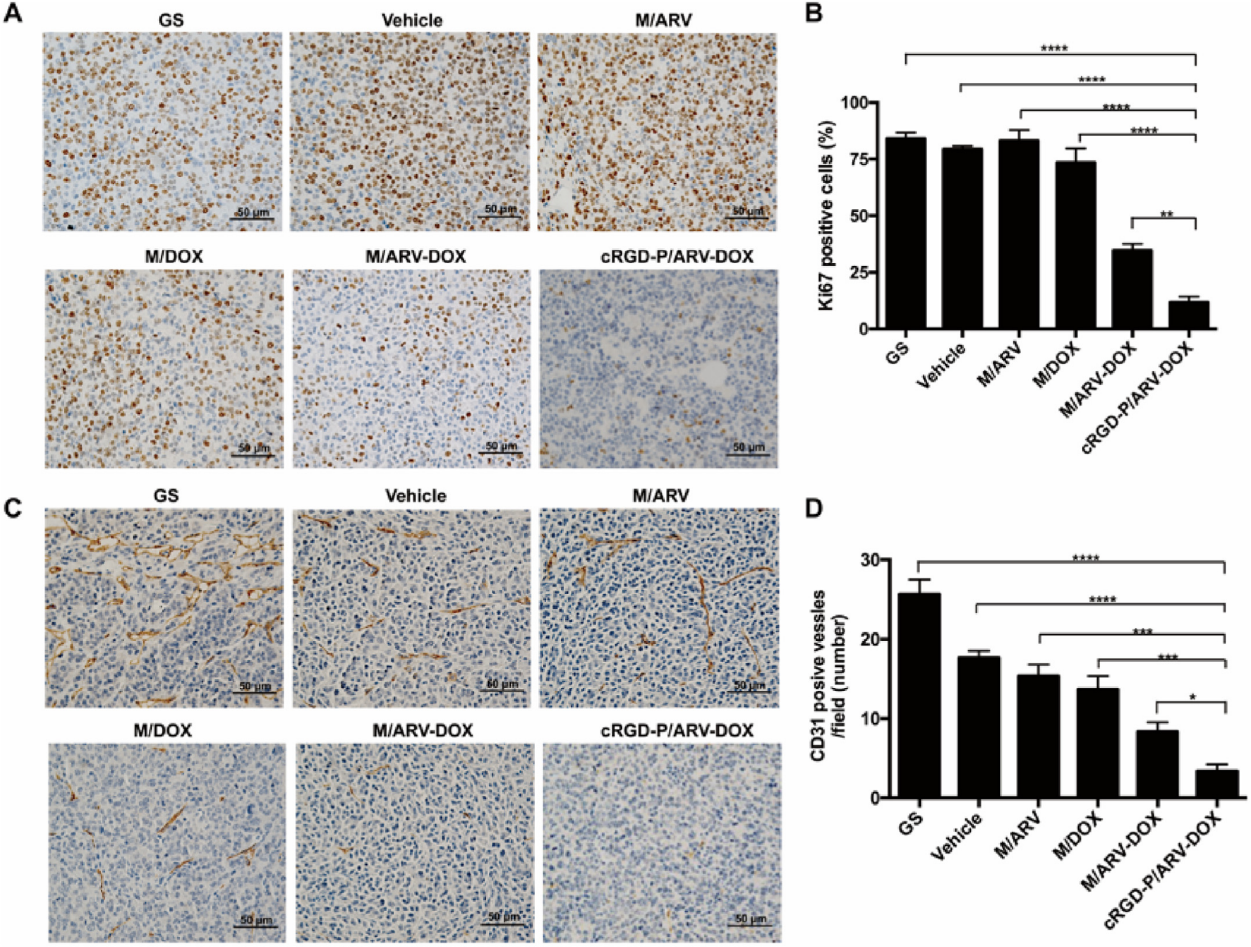
Combination therapy with DOX and ARV by cRGD-modified nanoparticle delivery inhibited tumor cell proliferation and vascular generation in a subcutaneous GL261 glioma model. (A) Ki67 staining was applied to detect tumor proliferation in tumor tissues of different groups. (B) The average percentage of Ki67-positive cells of each group was counted and analyzed. (C) CD31 staining was performed to visualize the tumor vascular generation. (D) The numbers of CD31-positive vessels in each group were counted and analyzed. Scale bars is 50 ​μm (n ​= ​5, ∗*p* ​< ​0.05, ∗∗*p* ​< ​0.01, ∗∗∗*p* ​< ​0.001, ∗∗∗∗*p* ​< ​0.001).

For exploration the effect of cRGD-P/ARV-DOX on tumor cell proliferation, the expression level of Ki67 by immunohistochemical staining was assessed in tumor tissues. In Fig. 7A and B, the group of cRGD-P/ARV-DOX showed fewer proliferating cells (Ki67 positive cells) than other control groups. The Ki67 positive rate in cRGD-P/ARV-DOX (11.77% ​± ​2.61%) was obvious lower than these in GS (84.16% ​± ​2.56%), Vehicle (79.43% ​± ​1.47%), M/ARV (83.23% ​± ​4.67%), M/DOX (73.39% ​± ​6.41%) and M/ARV-DOX (34.73% ​± ​2.90%) group. Moreover, the anti-angiogenic potential of these treatments were observed via immunohistochemical staining of CD31 in tumors. As shown in Fig. 7C and D, cRGD-P/ARV-DOX presented fewer microvessels numbers in the tumor segments compared with other control groups. The average number of microvessels of the cRGD-P/ARV-DOX group (3 per field) was lower than those in GS (26 per field), Vehicle (18 per field), M/ARV (15 per field), M/DOX (14 per field) and M/ARV-DOX (8 per field) group. Overall, these findings jointly demonstrated that cRGD-P/ARV-DOX could successfully improve antitumor effects by inhibiting tumor cell proliferation and vascular generation.

### *3.8* Safety assessment

The mice bodyweight surveillance, histological analysis of main organs and serological biochemical analysis in different groups were applied for evaluating the safety of the drugs. In Fig. 4, Fig. 5C, the body weight in all the treatment groups had no significant variation both in the subcutaneous model and in the orthotopic implantation model. Moreover, H&E staining of the vital organs were performed and no obvious abnormality of cRGD-P/ARV-DOX was found (Fig. S9). Furthermore, key serological biochemical indicators were also detected for safety evaluation, and values of these tests were within normal ranges (Fig. S10). Thereafter, a rudimentary safety assessment demonstrated the safety of the treatment in the animal experiment.

## 4 Discussion

The emergence of drug resistance has aggravated the difficulty of glioma therapy, making the efficacy of drug-resistant patients greatly reduced. The abnormal expression of BRD4 is thought to be related to cell proliferation and poor outcomes in some tumor types [36,37]. A high level of BRD4 protein has been significantly associated with the high sensitivity of NSCLC cells to BRD4 inhibitors [38]. Interestingly, we found that the expression of BRD4 was rapidly increased by cRGD-P/DOX within 12 ​h. Therefore, targeting BRD4 could be a credible way to conquer the DOX-based drug resistance.

Due to the presence of the BBB, many effective chemotherapeutic drugs fail to reach the glioma sites. Moreover, anti-cancer drugs are usually inefficient due to their poor aqueous solubility and non-specific toxicity to proliferating normal cells. In this study, we used cRGD-PEG-PLA to form core-shell micelles and to co-encapsulate DOX and ARV inside the hydrophobic core to treat glioma. Some studies showed that small micelles (in the sub-100 nm range) are easy to penetrate the BBB and accumulate in solid tumors [39]. In the current study, the prepared cRGD-P/ARV-DOX showed pleasant nano properties with a small size of 39.95 ​nm, which might be a good agent to go across the BBB and inside the tumor sites.

In this study, we found an increase in tumor cell death by blocking the cell cycle and promoting apoptosis when DOX and ARV were employed in combination. For tumor-bearing mice models, the combination therapy showed a significant inhibition of tumor growth and ARV also established an obvious sensitizing anti-tumor effect in tumor growth in vivo. Given the critical role of related protein in tumor progression, we, therefore, investigated the possible mechanisms of the combined treatment in vitro. As a transcription factor, STAT3 could promote growth and inhibit apoptosis of tumor cells by mediating transcription of downstream target genes, especially cyclin D1 [40]. In addition, cyclin D1 is demanded for the production and maintenance of tumor [41,42]. Recent studies have shown that inhibition of the activation of STAT3 could sensitize the effect of 5-FU by down-regulating cyclin D1 in colorectal cancer cells [43]. FOXO1 plays a key role in tumor growth inhibition by inducing growth arrest and apoptosis, while functional failure due to phosphorylation and proteasomal degradation has been related to cell malignancy [44]. Moreover, FOXO1 is a phosphorylated target of the protein kinase AKT. Inactivation of AKT causes up-regulated the FOXO1 expression, leading to inhibition of cell growth [45]. In the study, we verified that cRGD-P/ARV-DOX restrained the activation of STAT3 and AKT and induced the downregulation of p-FOXO1/FOXO1, cyclin D1 and CDK4. The initiator caspase-8, -9 and the executioner caspase-3 were identified as pivotal caspases in the apoptotic pathway relying on cleavage to activating caspases, which results cell apoptosis [46,47]. In many drug-resistant tumor cells, the increase of anti-apoptosis protein Bcl-2 and the decrease of pro-apoptotic protein Bax have been observed [48]. We found that treatment with cRGD-P/ARV-DOX promoted cell apoptosis via activating caspase cascade, downregulating Bcl-2 and upregulating Bax.

As expected, the cRGD-modified nanoparticles were able to enhance the anti-tumor efficacy in both an orthotopic implantation model and in a subcutaneous xenograft model compared to non-modified ones. This strategy reduces doses and achieves better curative effects, thereby decreasing the side effects on normal tissues. Moreover, to explore antitumor activity including apoptosis, proliferation and angiogenesis of glioma cells, TUNEL, Ki67 and CD31 staining were used in the study. When compared with cRGD-P/ARV, cRGD-P/DOX alone or non-targeted combination therapy, the co-delivery of cRGD-P/ARV-DOX significantly promoted tumor apoptosis, inhibited proliferation and decreased tumor angiogenesis. Furthermore, we assessed the biosafety of cRGD-P/ARV-DOX and observed no obvious systemic toxicity. Therefore, we demonstrate that the cRGD-P carrier has a pleasant biocompatibility and enhances the anti-cancer efficacy.

## 5 Conclusion

In conclusion, we verified that BRD4 was aberrantly overexpressed after treated with cRGD-P/DOX in glioma cells. The prepared cRGD-PEG-PLA nanoparticles were characterized as a targeted strategy for co-delivery of DOX and ARV to tumor cells. In vitro, cRGD-P/ARV-DOX micelles triggered G2/M-phase cell cycle arrest and promoted tumor cells apoptosis in glioma cells via suppressing the activation of STAT3 and AKT, downregulating p-FOXO1/FOXO1, cyclin D1 and CDK4, activating caspase cascade, downregulating Bcl-2 and upregulating Bax, resulting in obvious tumor suppression. Moreover, cRGD-P/ARV-DOX micelles augmented antitumor activities by enhancing anti-angiogenesis, inhibiting proliferation, and increasing apoptosis in vivo. Furthermore, the micelles showed a good safety profile. Therefore, the combination of DOX and ARV delivered by cRGD-modified nanoparticles might be a novel combination therapy for overcoming DOX-resistance cancers in the further clinic.

## Credit author statement

Yihong He: Conceptualization, Methodology, Investigation, Writing – original draft. Xin Zan: Conceptualization, Methodology, Investigation, Writing – original draft. Junming Miao: Conceptualization, Methodology, Investigation, Writing – original draft. Bilan Wang: Methodology, Investigation, Writing - original draft. Yin Wu: Methodology, Investigation. Yangmei Shen: Investigation. Xinchuan Chen: Conceptualization. Hongfeng Gou: Conceptualization. Songping Zheng: Methodology. Ning Huang: Supervision, Writing - review & editing. Yongzhong Cheng: Supervision, Writing - review & editing. Yan Ju: Writing - review & editing. Xianghui Fu: Writing - review & editing. Zhiyong Qian: Writing - review & editing. Supervision, Writing - review & editing. Peizhi Zhou: Supervision, Conceptualization, Writing - review & editing. Jiagang Liu: Conceptualization, Writing - review & editing, Project administration. Xiang Gao: Supervision, Conceptualization, Writing - review & editing.

## Declaration of competing interest

The authors declare that they have no known competing financial interests or personal relationships that could have appeared to influence the work reported in this paper.

## Data Availability

No data was used for the research described in the article.
