# Supplementary material for: Enhanced anti-glioma efficacy of doxorubicin with BRD4 PROTAC degrader using targeted nanoparticles
Source: Mater Today Bio. 2022 Sep 12;16:100423. doi: 10.1016/j.mtbio.2022.100423 (PMC9489811; doi:10.1016/j.mtbio.2022.100423)
Supplement: Multimedia component 1 [file mmc1.docx]

**Supporting information**

Enhanced anti-glioma efficacy of doxorubicin with BRD4 PROTAC degrader using targeted nanoparticles

Yihong He^a,b,1^, Xin Zan^a,1^, Junming Miao^a,1^, Bilan Wang^c^, Yin Wu^a^, Yangmei Shen^c^, Xinchuan Chen^a^, Hongfeng Gou^a^, Songping Zheng^a^, Ning Huang^b^, Yongzhong Cheng^a^, Yan Ju^a^, Xianghui Fu^a^, Zhiyong Qian^a^, Peizhi Zhou^a,^*, Jiagang Liu^a,^*, Xiang Gao^a,*^

*^a^Department of Neurosurgery and Institute of Neurosurgery, State Key Laboratory of Biotherapy and Cancer Center, West China Hospital, West China Medical School, Sichuan University and Collaborative Innovation Center for Biotherapy, Chengdu 610041, China*

*^b^Department of Pathophysiology, West China College of Basic Medical Sciences & Forensic Medicine, Sichuan University, 610041 Chengdu, China*

*^c^West China Second University Hospital of Sichuan University, Chengdu, 610041, PR China.*

^#^These authors made equal contributions to this work.

* Corresponding author: Peizhi Zhou, Jiagang Liu and Xiang Gao

Tel.: +86 28 8542 2136;

Fax: +86 28 8550 2796.

Email addresses: [peizhizhouns@126.com](mailto:peizhizhouns@126.com) (Peizhi Zhou**)**, jiagang_liu@163.com (Jiagang Liu) and [xianggao@scu.edu.cn](mailto:xianggao@scu.edu.cn) (Xiang Gao)

**Supplementary materials and methods**

**Chemicals and materials**

MPEG_2000_, DL-lactide, PI, DMSO, 3-(4,5- dimethylthiazol-2-yl)-2,5-diphenyltetrazolium bromide (MTT), Tin (Ⅱ)-ethylhexanoate (Sn (Oct)_2_), PLA_2000_-COOH, N, N-di-isopropylcarbodiimide (DIC), HOOC-PEG_2000_-NH_2_, N-methylpyrrolidone (NMP), dichloroethane (EDC), N-hydroxysulfosuccinimide (NHS) were purchased from Sigma-Aldrich (St. Louis, MO, USA). Cyclo (Arg-Gly-Asp-D-Phe-Lys) (cRGDfk) was purchased from NJpeptide Biology Technology Co., Ltd (China). Doxorubicin hydrochloride (DOX) was purchased from Dalian Meilun Biology Technology Co., Ltd (China). ARV-825 was purchased from MedChemExpress (USA). The Annexin V-FITC/PI Apoptosis Detection Kit was purchased from BD Biosciences (USA). The TUNEL Detection Kit were purchased from Promega (USA). Goat anti-mouse Ki67 antibody and goat anti-mouse CD31 antibody were purchased from servicebio (China). Hematoxylin and eosin (H&E) and RIPA buffer were purchased from Beyotime (China). Protease inhibitor cocktail was purchased from Mei5bio (China). 12.5% SDS-PAGE was purchased from Baihe Science & Technology Co. (China). PVDF membrane was purchased from Merk Millipore (Germany). Antibody against BRD4 was purchased from abcam (UK). Antibodies against STAT3, p-STAT3, cyclin D1, CDK4, AKT, p-AKT, FOXO1, p-FOXO1, caspase 3, cleaved caspase 3, caspase 8, cleaved caspase 8, caspase 9, cleaved caspase 9, Bcl-2, Bax, β-actin, and GAPDH were purchased from Cell Signaling Technology (USA).

**Cells and animals**

The GL261 (murine glioma cell line) and U251 (human glioma cell line) cells were obtained from the American Type Culture Collection (ATCC). They were maintained in DMEM (Nanjing BioChannel Biotechnology Co., Ltd, China) including 10% fetal bovine serum (Gibco, USA), 100 U/mL penicillin and 100 μg/mL streptomycin, culturing at 37 °C in a 5% CO_2_ incubator. C57BL/6 mice were acquired from Huafukang (Beijing, China) and kept under SPF conditions. All animal experiments were approved by the Animal Experimental Ethics Committee of the State Key Laboratory of Biotherapy (SKLB), Sichuan University.

**Western blot**

GL261 cells were treated with cRGD-P/DOX (0.5 μg/ml) for 0, 2, 4, 8 and 12 h. Additionally, they were incubated with cRGD-P/ARV, cRGD-P/DOX and cRGD-P/ARV-DOX at 0.0625 μg/ml for 24 h, respectively. U251 cells were incubated with cRGD-P/ARV, cRGD-P/DOX and cRGD-P/ARV-DOX at 0.125 μg/ml for 48 h, respectively. Cells were lysed in RIPA buffer and protease inhibitor on ice for 30 minutes, following centrifuged at 12,000 rpm for 15 minutes at 4°C. The supernatants were collected as the total protein of cells. The same amount of total protein from each group was fractionated by a 12.5% SDS-PAGE. Then, the samples on the gel were transferred to PVDF membranes. The membranes were blocked by 5% nonfat dry milk, and then incubated with primary antibodies. The membranes were reacted with HRP-conjugated secondary antibodies. Finally, the membrane was visualized by a chemiluminescence imaging system (CLINX, China).

**Supplementary Figures**

**
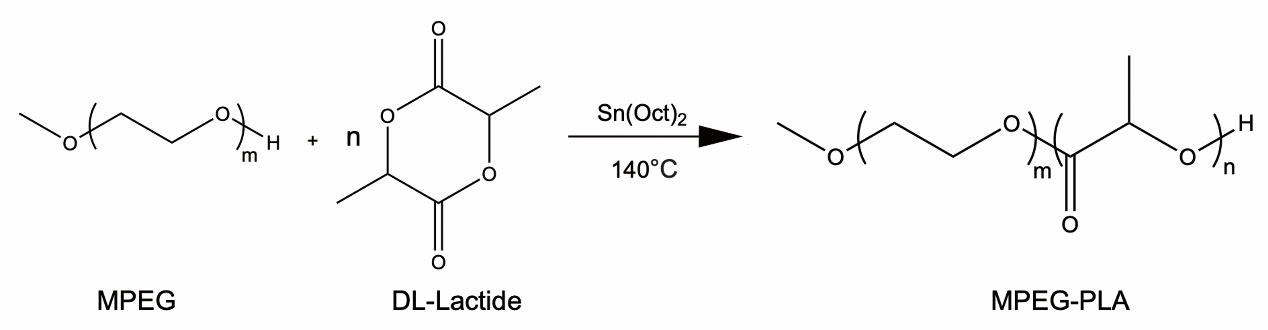
**

**Fig. S1. Synthesis route of MPEG-PLA.**

**
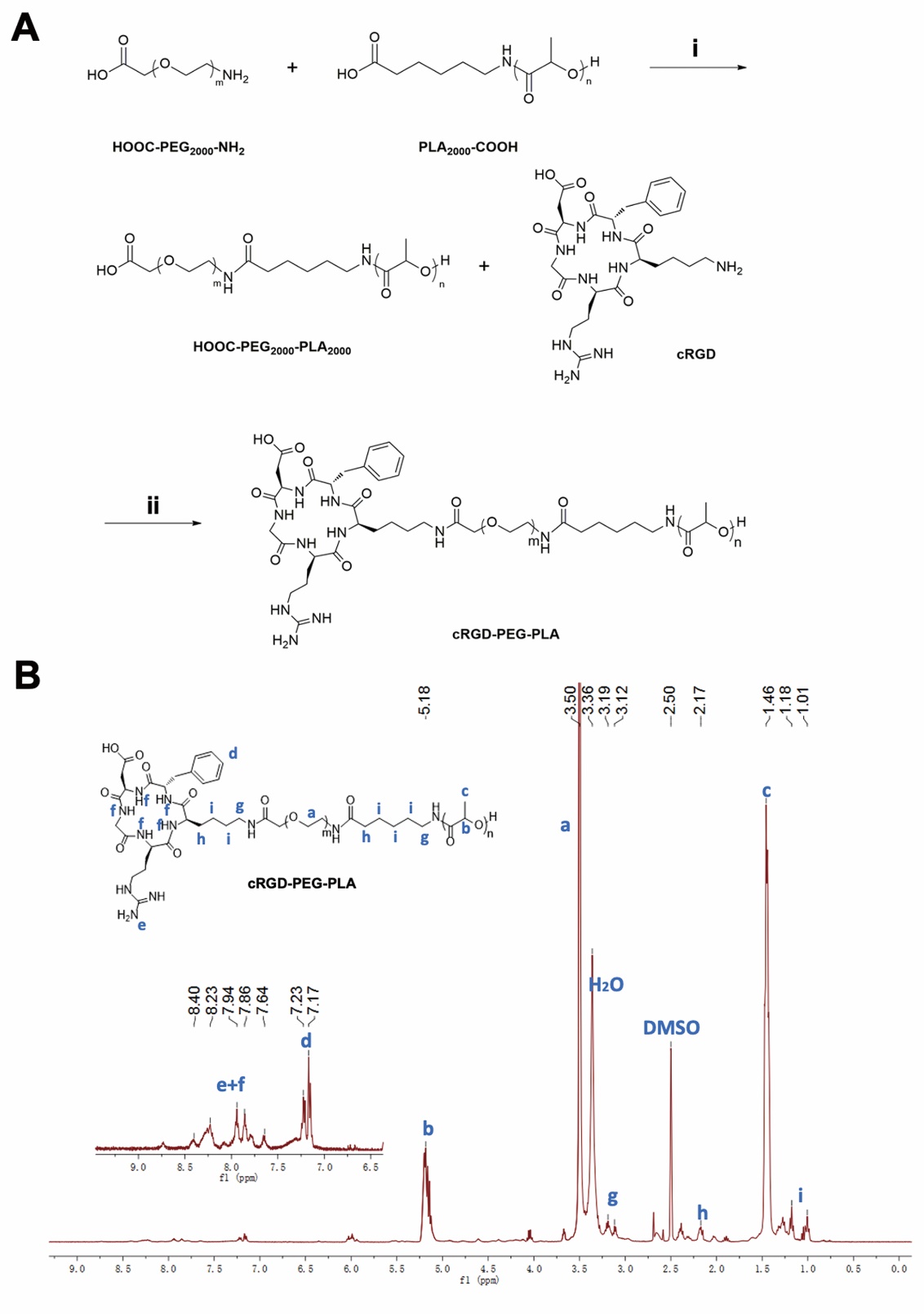
**

**Fig. S2. Synthesis procedure and structural identification of cRGD-PEG-PLA.** (A) Conditions: (i) DIC, triethylamine, r.t., 6h; (ii) EDC, NHS, triethylamine, r.t., overnight. (B) Chemical shifts of cRGD-PEG-PLA are shown by the δ scale, which is referenced from the protium of the solvent (DMSO: δ 2.50) for ^1^H NMR.


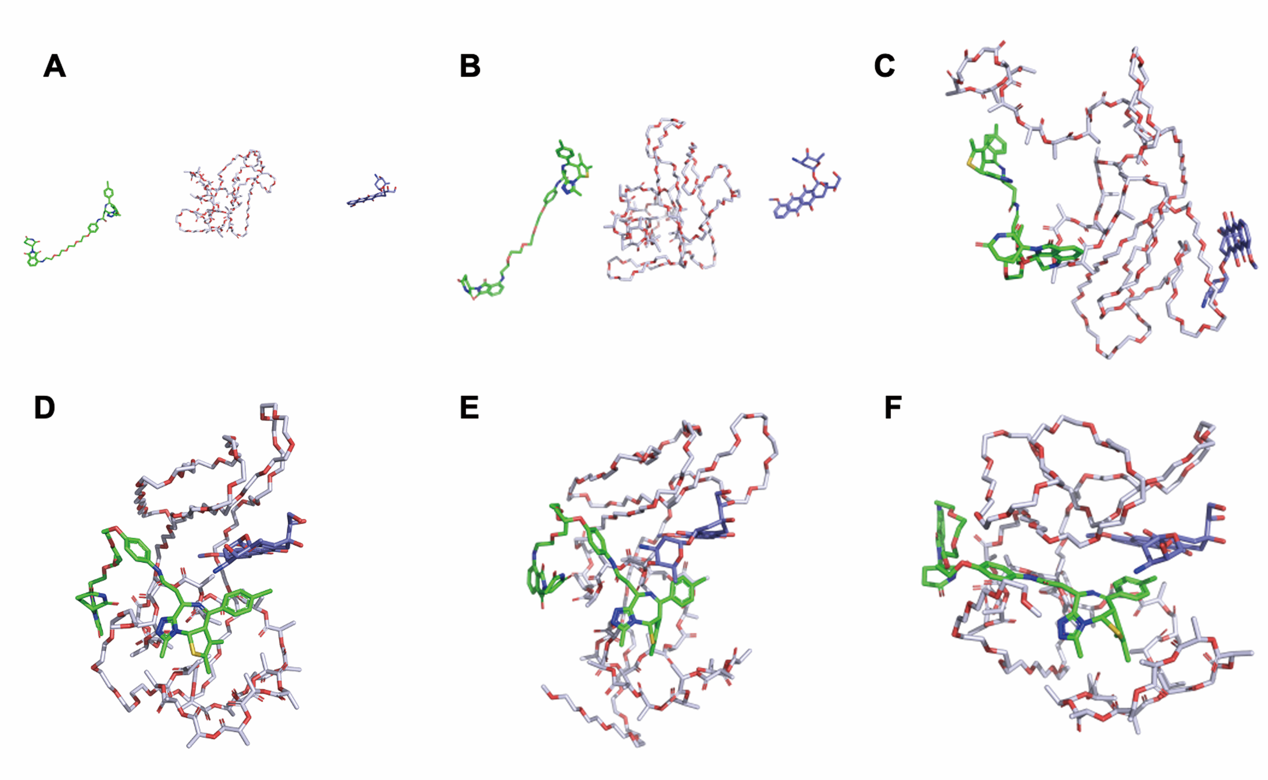


**Fig. S3.** **Computational simulation analysis.** Interaction modes among the cRGD-PEG-PLA, ARV-825 and DOX revealed by Langevin dynamics simulation in the water environment (pH = 7.0). The conformations (A), (B), (C), (D), (E) and (F) were corresponded to the snapshots of this complex at 0 ns, 2 ns, 4 ns, 6 ns, 8 ns和10 ns, respectively.


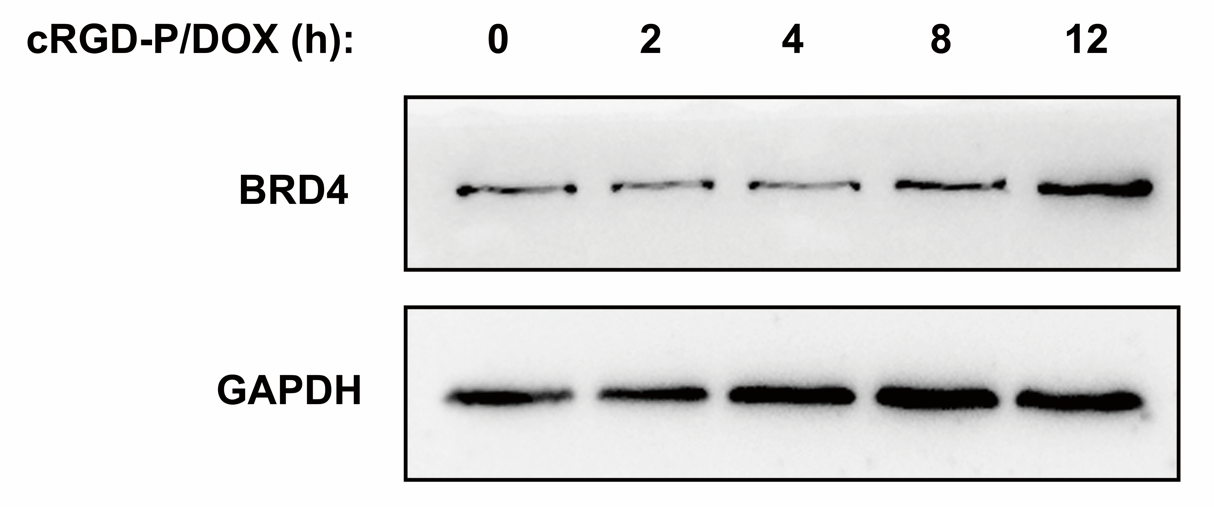


**Fig. S4. BRD4 expression analysis.** Western blotting was performed to detect the protein levels of BRD4 in GL261 cells treated with cRGD-P/DOX (0.5 μg/ml) for 0, 2, 4, 8 and 12 h.


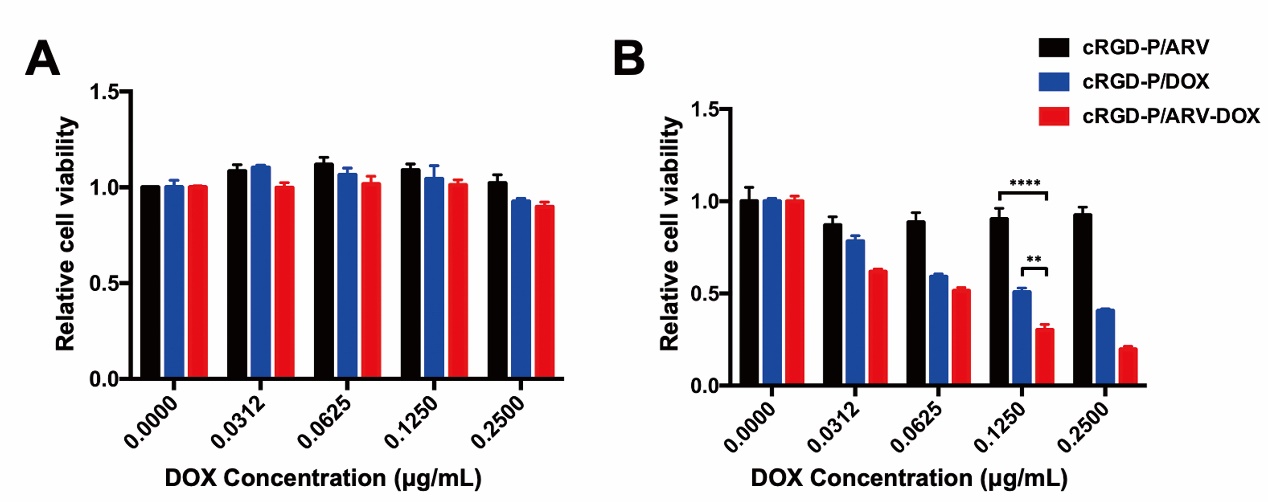


**Fig. S5. Cell viability study of the nanocomposites by MTT assay.** U251 cells were incubated with cRGD-P/ARV, cRGD-P/DOX and cRGD-P/ARV-DOX at different concentration from 0 μg/ml to 0.25 μg/ml for 24 h (A) and 48 h (B) (n = 3, **p<0.01, ****p<0.0001).


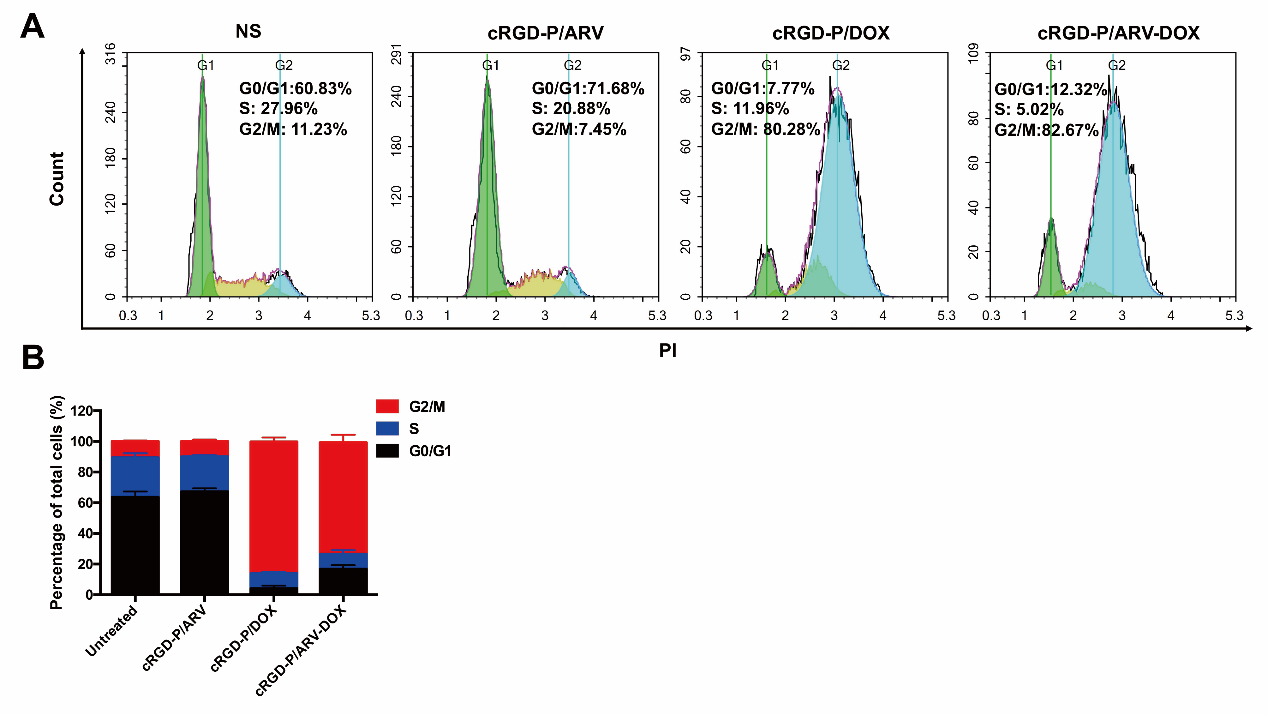


**Fig. S6. Analysis of cell cycle in U251 cells.** U251 cells were treated with cRGD-P/ARV, cRGD-P/DOX and cRGD-P/ARV-DOX at 0.125 μg/ml for 48h. (A) Cells were collected and stained with PI for flow cytometric analysis. (B) Populations of cells at G0/G1, S and G2/M phases were collected as percentages of the whole cell population.

**
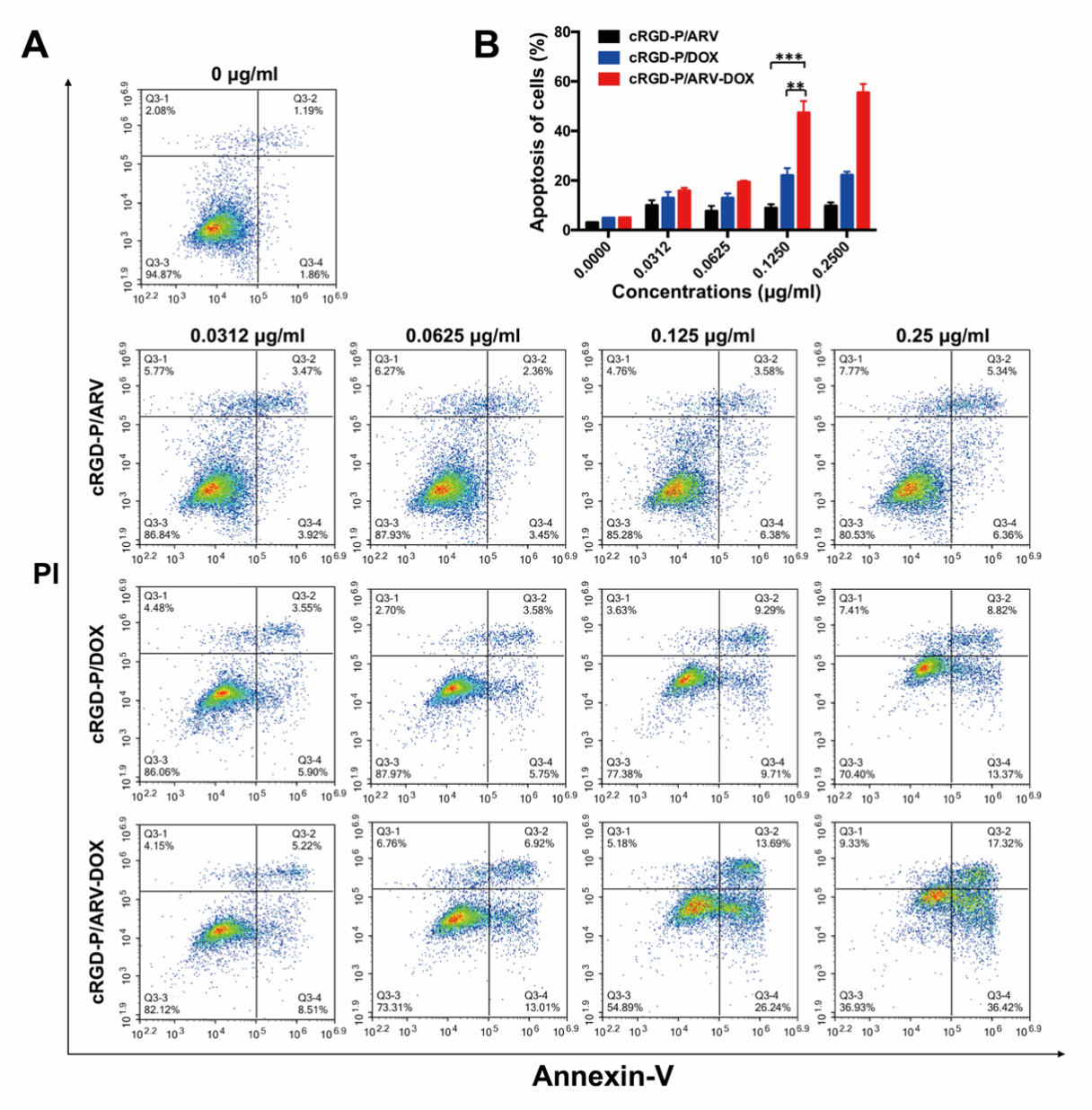
**

**Fig. S7. Apoptosis assay of U251 cells.** U251 cells were treated with cRGD-P/ARV, cRGD-P/DOX and cRGD-P/ARV-DOX at different concentration (0 μg/ml to 0.25 μg/ml) for 48 h. (A) and (B) Cells were collected and stained with Annexin-V and PI for flow cytometric analysis (n=3, **p<0.01, ***p<0.001).


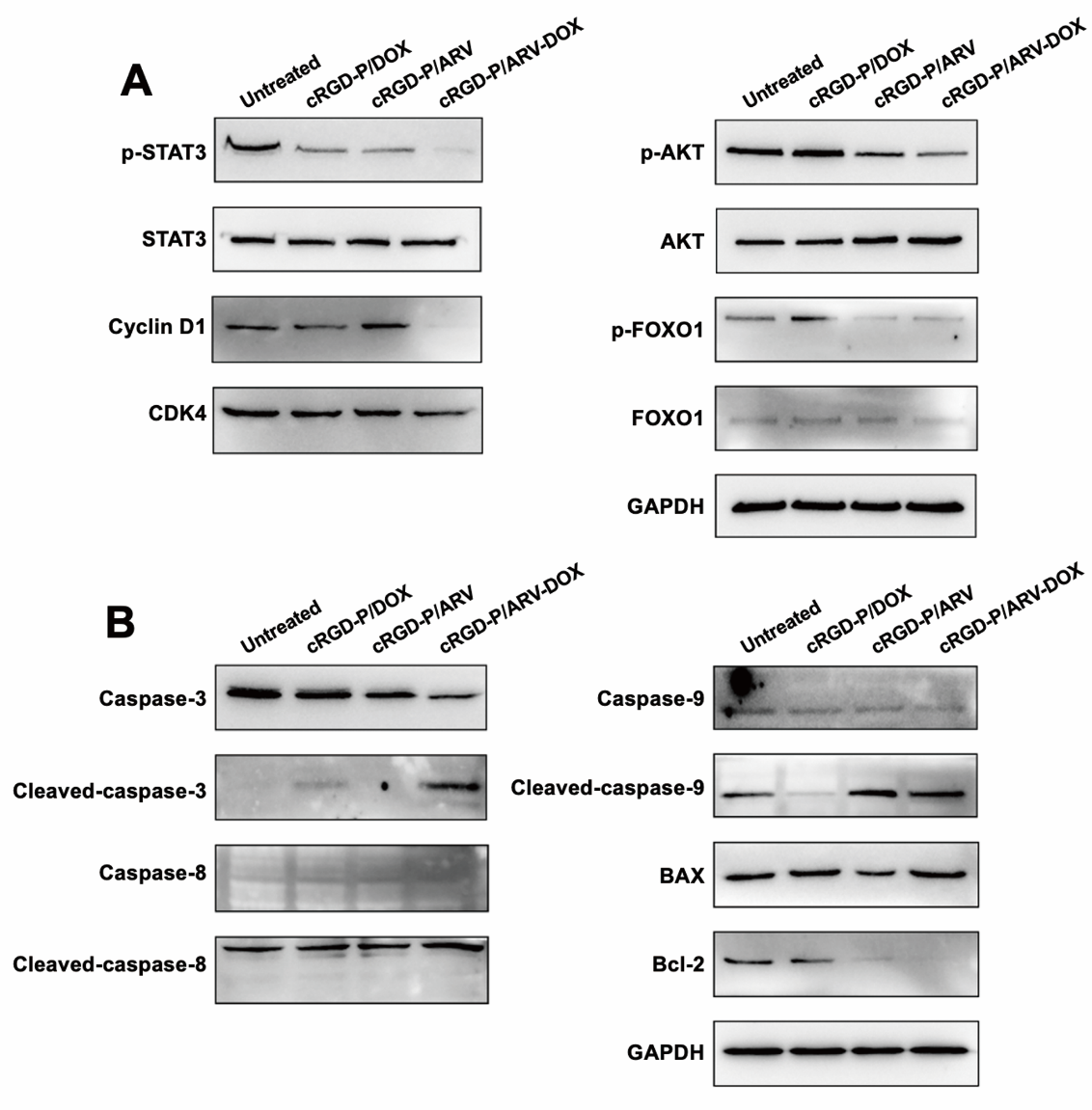


**Fig. S8. Western blot analysis.** After being treated with NS, cRGD-P/ARV (0.125 μg/ml), cRGD-P/DOX (0.125 μg/ml) and cRGD-P/ARV-DOX, U251 cells were collected for proteins expression analysis. (A) Cell proliferation and cycle-related proteins. (B) Apoptosis-related proteins. All gene expressions were normalized to GAPDH (reference gene).


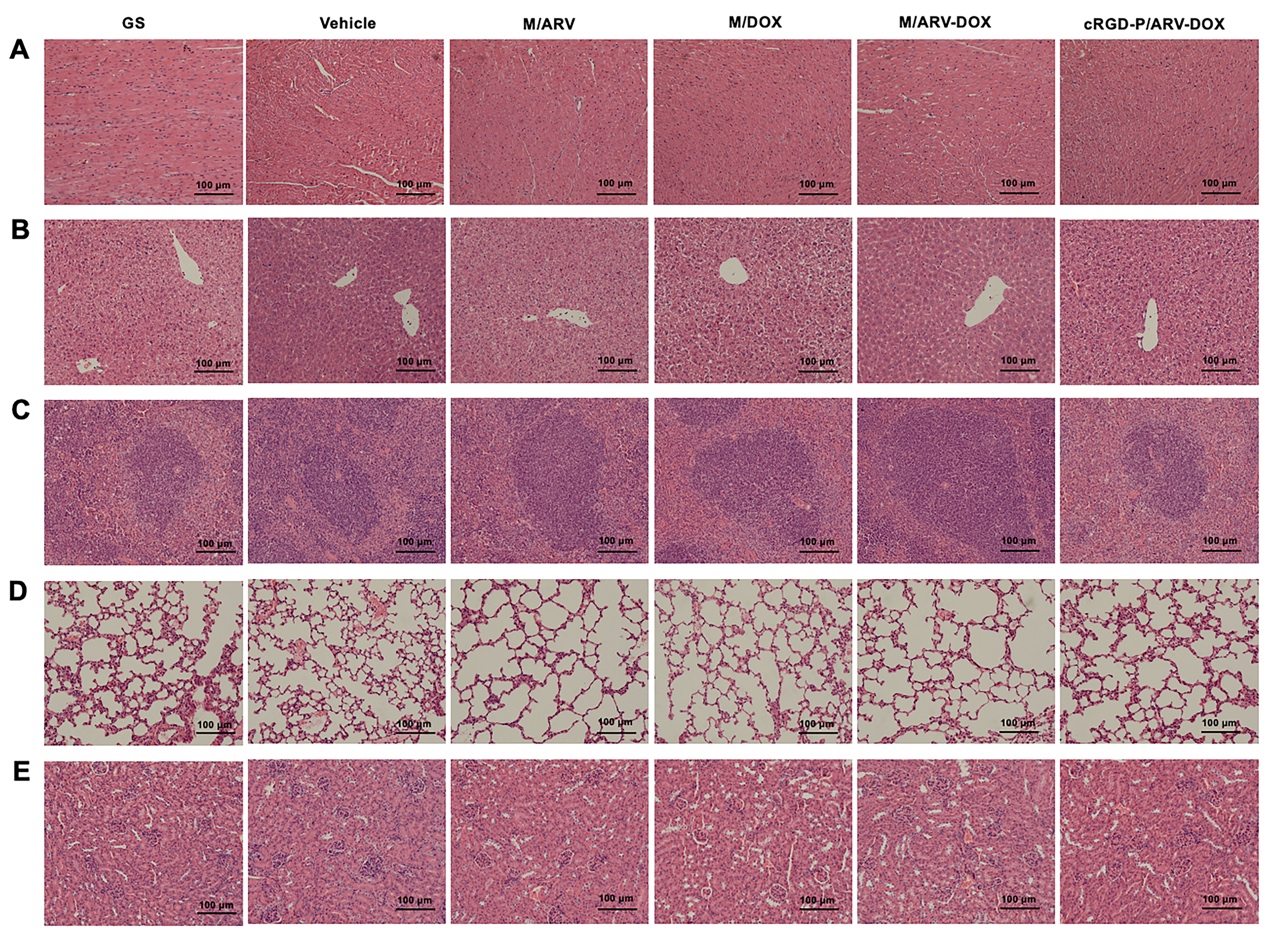


**Fig. S9. Toxicity assessment in H&E staining with pathological section.** HE-stained vital organ sections were from subcutaneous model for histological examinations. (A) heart, (B) liver, (C) spleen, (D) lung, and (E) kidney. Scale bars is 100 μm, n=5.


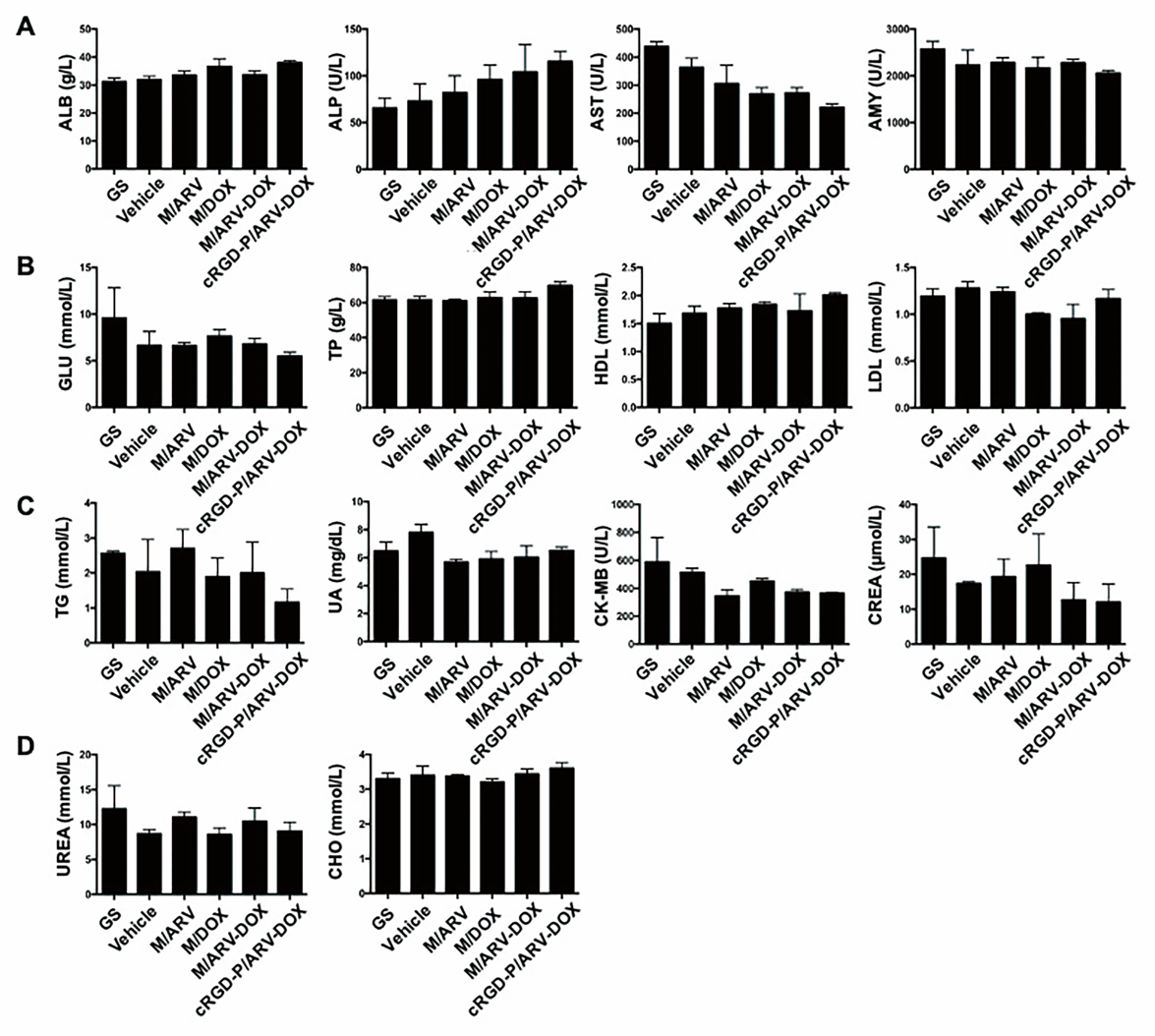


**Fig. S10. Serologic biochemical analyses.** Serologic biochemical detection of GS, Vehicle, M/ARV, M/DOX, M/ARV-DOX or cRGD-P/ARV-DOX treated mice included: (A) ALB, albumin; ALP, alkaline phosphatase; AST, aspartate aminotransferase; AMY, amylase; (B) GLU, glucose; TP, total protein; HDL, high-density lipoprotein-cholesterol; LDL, low-density lipoprotein-cholesterol; (C) TG, triglycerides; UA, uric acid; CK-MB, creatine kinase MB; CREA, creatinine; (D) UREA, urea; CHO, total cholesterol. n=3.
